# Characterization of novel biomarkers in selecting for subtype specific medulloblastoma phenotypes

**DOI:** 10.18632/oncotarget.6195

**Published:** 2015-10-20

**Authors:** Lisa Liang, Christopher Aiken, Robyn McClelland, Ludivine Coudière Morrison, Nazanin Tatari, Marc Remke, Vijay Ramaswamy, Magimairajan Issaivanan, Timothy Ryken, Marc R. Del Bigio, Michael D. Taylor, Tamra E. Werbowetski-Ogilvie

**Affiliations:** ^1^ Regenerative Medicine Program, Department of Biochemistry and Medical Genetics, University of Manitoba, Winnipeg, Manitoba, Canada; ^2^ Department of Immunology, University of Manitoba, Winnipeg, Manitoba, Canada; ^3^ Arthur and Sonia Labatt Brain Tumour Research Centre and Program in Developmental and Stem Cell Biology, The Hospital for Sick Children, Toronto, Ontario, Canada; ^4^ Cancer Care Manitoba (CCMB), Winnipeg, Manitoba, Canada; ^5^ Department of Neurosurgery, University of Kansas, Kansas City, Kansas, USA; ^6^ Department of Pathology, University of Manitoba and Manitoba Institute of Child Health, Winnipeg, Manitoba, Canada

**Keywords:** medulloblastoma, biomarkers, progenitors, self-renewal, high-throughput flow cytometry

## Abstract

Major research efforts have focused on defining cell surface marker profiles for characterization and selection of brain tumor stem/progenitor cells. Medulloblastoma is the most common primary malignant pediatric brain cancer and consists of 4 molecular subgroups: WNT, SHH, Group 3 and Group 4. Given the heterogeneity within and between medulloblastoma variants, surface marker profiles may be subtype-specific. Here, we employed a high throughput flow cytometry screen to identify differentially expressed cell surface markers in self-renewing vs. non-self-renewing SHH medulloblastoma cells. The top 25 markers were reduced to 4, CD271/p75NTR/NGFR, CD106/VCAM1, EGFR and CD171/NCAM-L1, by evaluating transcript levels in SHH tumors relative to samples representing the other variants. However, only CD271/p75NTR/NGFR and CD171/NCAM-L1 maintain differential expression between variants at the protein level. Functional characterization of CD271, a low affinity neurotrophin receptor, in cell lines and primary cultures suggested that CD271 selects for lower self-renewing progenitors or stem cells. Moreover, CD271 levels were negatively correlated with expression of SHH pathway genes. Our study reveals a novel role for CD271 in SHH medulloblastoma and suggests that targeting CD271 pathways could lead to the design of more selective therapies that lessen the broad impact of current treatments on developing nervous systems.

## INTRODUCTION

Medulloblastoma (MB) is the most common malignant primary pediatric brain tumor. Primary tumors typically develop in the cerebellum and fourth ventricle; however, extensive dissemination through the cerebrospinal fluid often leads to metastasis and tumor recurrence [1]. Despite improved clinical outcomes and a 5 year survival rate of 60-70% [1], children with MB often suffer from cognitive and physical dysfunction resulting from current treatment of resection, followed by the long-term toxicities associated with chemotherapy and radiation [2].

Historically, MB was divided into subtypes based on morphology and histological characteristics including variants such as, desmoplastic, classic, large cell anaplastic, and medulloblastoma with extensive nodularity [3]. Due to advances in genomic sequencing and microarray technology, MB has now been classified into 4 distinct molecular subtypes based on genomic alterations, gene expression patterns and response to treatment: WNT, SHH, Group 3 and Group 4 [3]. WNT and SHH tumors are characterized by activation of the WNT and SHH signaling pathway respectively and are associated with a more favorable to intermediate prognosis [3]. However, within the SHH subgroup, those exhibiting *TP53* mutations are associated with poor outcome [4]. Less is known about the molecular basis of disease progression for the most aggressive Group 3 tumors that exhibit the worst prognosis as well as Group 4 MBs.

Currently, the majority of studies on the 4 MB variants focus on mutation analysis and differential gene expression [5-7]. While this work has revolutionized our understanding of pediatric brain tumor heterogeneity, the specific functional role of mutated and differentially expressed genes is not always understood and will likely have to be considered in a subtype specific manner. Understanding how these genes contribute to cellular heterogeneity will also provide a more complete picture of disease complexity.

Cancer stem cell (CSC) theory has been employed to explain the cellular heterogeneity within a variety of cancers including MB [8]. This theory poses that some cancers contain a subpopulation of cells (CSCs) that exhibit stem cell-like properties. These properties include the ability to self-renew or maintain themselves indefinitely in a primitive state and undergo multi-lineage differentiation [9]. CSCs are not necessarily rare but are believed to be responsible for tumor initiation and/or maintenance in a variety of cancers.

The existence of brain tumor CSCs, also known as brain tumor propagating cells (BTPC), was first demonstrated by Singh et al., using the cell surface marker CD133 to select for a cell population showing increased self-renewal in glioblastoma and medulloblastoma both *in vitro* and *in vivo* [10, 11]. While CD133 is the most commonly utilized BTPC marker, recent studies have shown that even CD133− cells exhibit self-renewal capacity and can generate highly aggressive tumors *in vivo* [10-12]. This is complicated by the fact that CD133 is not exclusive to tumor propagating cell populations and is also expressed in normal stem cells and a variety of differentiated epithelial cells [12]. In addition, CD15/SSEA1 (Stage Specific Embryonic Antigen-1) has also been shown to select for cells that have tumorigenic capacity in a *Ptch* mutant mouse model of SHH MB [13, 14]. Read et al. [13] demonstrated that tumors are not propagated by a stem-like CD133+ population but by cells marked by the neuronal progenitor markers Math1 and CD15. Ward et al. also demonstrated the tumorigenic capacity of CD15+ cells from *Ptc*+/− mice; however, these authors suggested that the CD15+ population represents a stem-like rather than a progenitor cell phenotype [13, 14]. More recently, the neural stem cell marker Sox2 has been shown to play a role in SHH MB tumor propagation [15, 16]. Vanner et al [15] showed that the Sox2+ cell population was enriched following treatment with chemotherapy and SHH antagonists, resulting in tumor growth and relapse.

Although the SHH variant is associated with an intermediate prognosis and a 5-year survival rate of 60-80% [3], recent studies have demonstrated heterogeneity within the subtype [4, 17]. In addition, the cell of origin for SHH MB is still under debate [13-15]. Identification of additional cell surface markers that select for stem and/or progenitor cells will be necessary to further delineate the cellular complexity within these tumors. Given the heterogeneity observed between and even within the MB variants, these signatures may also be distinctly associated with a particular subtype.

Identification of surface markers capable of enriching for TPCs in a cancer is generally achieved using flow cytometry with a small number of single antibodies selective for surface markers already known to play a role in normal stem cell biology [11, 13, 14, 18]. This practice has major bias towards the surface markers selected and does not cover a large majority of known human cell surface markers. A more efficient, less biased system capable of screening high numbers of surface markers is necessary. To this end, we employed a high throughput flow cytometry screening platform to identify additional cell surface markers associated with stem/progenitor cell phenotypes specifically in the SHH molecular variant. This platform has recently been introduced as a mechanism for identifying primary *vs*. metastatic colon cancer cell lines [19], for distinguishing cells at various stages of neural lineage specification [20], and most recently, for identification of adhesion receptors contributing to glioblastoma self-renewal and tumor growth [21]. Further functional characterization of CD271 was performed as proof of principle to demonstrate the power this unique approach has in identifying novel markers associated with the SHH MB subtype. CD271, also known as the p75 neurotrophin receptor (p75NTR) or nerve growth factor receptor (NGFR), is a transmembrane glycoprotein that plays a variety of roles in normal neurodevelopment including growth cone elongation, axon guidance, cell survival and cell death [22]. Our results suggest that CD271 selects for a lower self-renewing stem/progenitor cell phenotype specifically in SHH medulloblastoma and further underscore the cellular heterogeneity in these tumors.

## RESULTS

### High throughput flow cytometry screens and MB transcriptome datasets reveal 4 cell surface markers that are differentially expressed between self-renewing *vs*. non-self-renewing SHH MB tumorspheres and SHH *vs*. the other MB variants

Utilizing a BD Lyoplate™ 242 human cell surface marker panel, we screened for differential expression in self-renewing (continue to form tumorspheres over subsequent passage) *vs*. non-self-renewing (gradually lose tumorsphere forming capacity and become adherent) Daoy MB subclones that were previously derived by single cell sorting and expansion from parental cultures [18]. The Daoy MB cell line is derived from a desmoplastic MB [23], has been shown to exhibit global activation of SHH-pathway genes [24-26] and is statistically classified as SHH subgroup based on hierarchical clustering and PCA analysis with patient samples [27]. This cell line continues to be utilized as an alternative or supplement to working with fresh patient tissue or minimally cultured samples [24, 28, 29], as it has been very difficult to establish cultures from primary MB tumors and maintain them over subsequent passage. Using self-renewing and non-self-renewing Daoy tumorspheres, we conducted 2 independent screens and the results were reproducible between trials. Twenty-five markers were found to be differentially expressed between the phenotypes, with more than a 2 fold difference in frequency and mean fluorescence intensity (Table 1 and Figure S1).

**Table 1:** Twenty-five differentially expressed cell surface markers in self-renewing *vs*. non self-renewing Daoy tumorspheres using the BD Lyoplate ™ Human Cell Surface Marker Screening Panel

Cell Surface Marker	Self-renewing (SR) Tumorspheres		Non Self-renewing (SR) Tumorspheres		Fold change in Self-renewing vs. non-self-renewing Tumorspheres	
	% Positive	MFI	% Positive	MFI	Fold change % Positive	Fold change MFI
**Disialoganglioside GD2**	21.250	5.631	0.247	0.988	85.911	5.700
**CD227/MUC1**	16.795	2.283	1.939	1.114	8.664	2.048
**CD271/p75NTR**	23.450	2.754	3.130	1.189	7.492	2.317
**CD57/HNK1**	90.050	19.990	28.500	2.270	3.160	8.804
**CD97**	16.550	2.230	43.000	4.927	−2.598	−2.210
**SSEA-4**	16.450	1.632	49.200	4.502	−2.991	−2.759
**CD70**	18.800	1.186	58.650	3.073	−3.120	−2.591
**CD71/TFRC**	9.975	1.841	38.150	4.476	−3.825	−2.431
**CD39/ENTPD1**	4.205	1.458	17.155	4.279	−4.080	−2.936
**CD106/VCAM1**	14.500	2.041	73.750	34.876	−5.086	−17.086
**TRA-1-60**	2.400	0.982	13.200	2.690	−5.500	−2.740
**CD171/NCAM-L1**	5.445	1.487	40.400	4.608	−7.420	−3.100
**CD10/CALLA**	3.000	1.242	22.550	2.537	−7.517	−2.042
**CD326**	1.096	0.737	9.760	2.332	−8.909	−3.163
**CD107b/LAMP2**	2.315	1.048	22.000	3.845	−9.503	−3.668
**CD86**	2.515	1.227	25.200	2.844	−10.020	−2.318
**EGF-r**	2.670	1.392	38.400	4.833	−14.382	−3.471
**CD108/Sema7a**	4.495	1.446	67.650	10.332	−15.050	−7.146
**Hem. Prog. Cell**	1.071	1.177	17.540	2.426	−16.377	−2.061
**MIC A/B**	1.532	1.161	26.250	3.528	−17.140	−3.040
**CD107a/LAMP1**	1.710	1.143	29.900	5.384	−17.485	−4.711
**CD274/PD-L1**	1.835	1.344	35.000	3.721	−19.074	−2.769
**CD119/IFNGR1**	2.100	1.489	40.650	3.050	−19.357	−2.049
**CD130**	0.835	1.206	24.600	2.421	−29.461	−2.007
**CD273/PD-L2**	0.566	0.970	27.450	2.810	−48.498	−2.896

MFI: Mean Fluorescence Intensity

**Figure 1:**
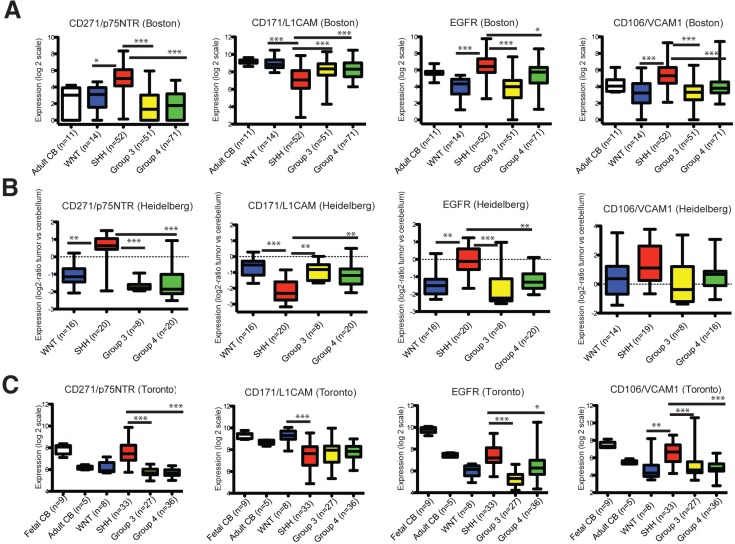
Transcript levels of CD271/p75NTR, CD171L1CAM, EGFR and CD106/VCAM1 across the 4 MB molecular variants Note that the CD271 Toronto dataset was previously published in Neoplasia, 15, Morrison et al., *Deconstruction of Medulloblastoma cellular heterogeneity reveals differences between the most highly invasion and self-renewing phenotypes*, 384-398, Copyright Elsevier (2013). **A.**-**C.** Gene expression data from 3 independent transcriptome datasets representing 548 patient samples showing downregulation of CD171/NCAM-L1 and relative enrichment of CD271/p75NTR, CD106/VCAM1, and EGFR, in SHH tumors compared with the other variants. Bars denote 1.5 interquartile range within each group. All subgroups were compared using a Kruskal-Wallis test for significance. Data are presented as log2 -transformed signal intensity. *P* < 0.05*, *P* < 0.01**, *P* < 0.001***.

We next determined whether any of the 25 candidate cell surface markers also exhibit differential expression in SHH patient samples relative to samples from the other MB subtypes. Three independent transcriptome datasets derived from gene expression profiling of MB samples across multiple centers, patient populations and technical platforms that together represent 548 patient samples were interrogated [30-32]. Most candidates exhibited differential expression across subtypes in 1 or 2 datasets. However, 4 of the 25 cell surface markers displayed consistent and differential expression in SHH MB compared to WNT, Group 3, and Group 4 MB patient samples across all 3 datasets. CD271, CD106/VCAM1, and EGFR were upregulated; whereas, CD171/NCAM-L1 was downregulated in SHH MB relative to the other variants (Figure 1A-1C; Figure S2).

### CD271 and CD171 are differentially expressed in MB cell lines/primary cultures and patient samples at the protein level

**Figure 2:**
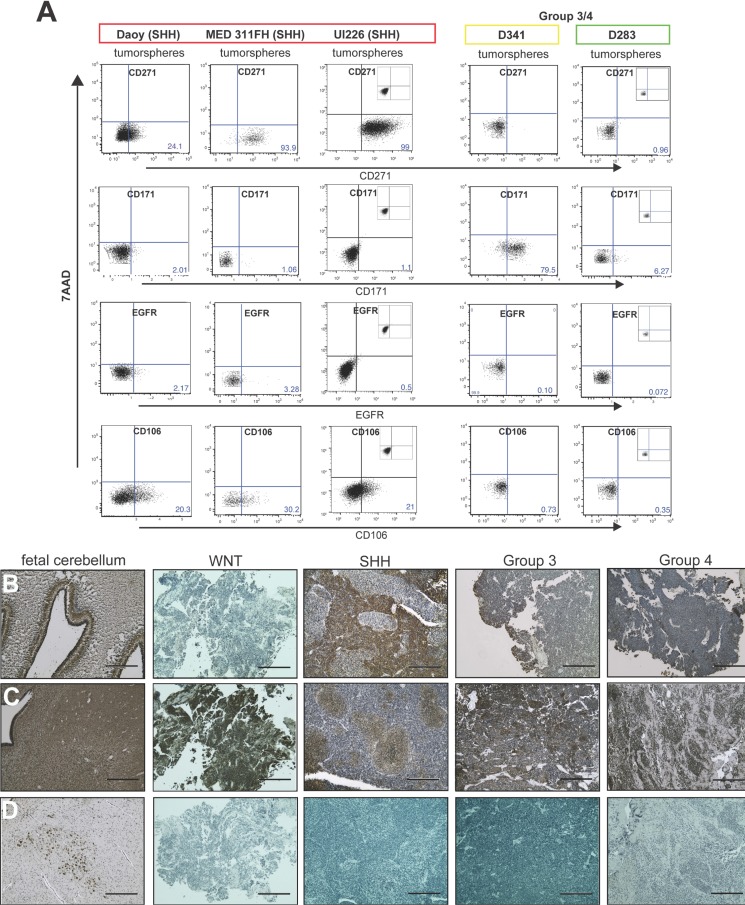
Candidate cell surface markers are differentially expressed in MB cell lines/primary cultures and patient samples **A.** Representative dot plots of staining for candidate biomarkers in Daoy, MED311-FH and UI226 tumorspheres (SHH variant) *vs*. tumorspheres from Group 3/Group 4 cell lines by flow cytometry. Insets: respective isotype controls. 7AAD: 7-aminoactinomycin D cell viability dye. **B.**-**D.** CD271 (B), CD171 (C) and CD106 (D) expression in paraffin embedded sections of fetal cerebellum and primary medulloblastoma samples. Scale bar: 400 μm.

We next evaluated expression levels of these 4 markers in MB tumorspheres from a variety of cell lines by flow cytometry. In addition to Daoy, we utilized the recently derived MED 311-FH SHH cell line and UI226 low passage primary cultures that have been subtyped by nanoString as previously described [33] and designated SHH. Low passage primary cultures, which are more clinically relevant, provide an excellent complementary model to cultured cell lines such as Daoy. D341 [34] is a Group 3 MB, and D283 [35] has recently been classified as Group 4 [36]; however, previous studies have demonstrated that D283 also exhibits features of Group 3 such as high c-myc levels [37]. To our knowledge, there are no WNT MB cell lines; thus, we used both D341 and D283 as representative non-SHH variant cells. Based on the gene expression profiling results, we predicted that CD271, CD106 and EGFR would be higher and CD171 lower in Daoy, MED 311 and UI226 relative to D341 and D283 tumorspheres. Indeed, CD271, CD106 and EGFR are higher and CD171 is lower in Daoy, MED311 and UI226 *vs*. D341 and D283 tumorspheres (Figure 2A). However, EGFR levels were quite low across all cell lines, and we did not pursue this marker further (Figure 2A).

Immunohistochemical staining of paraffin embedded sections from primary MB patient samples also revealed differential CD271 and CD171 expression patterns between the molecular variants (Figure 2B-2D). Specifically, CD271 levels were higher in SHH MB samples and in the external granular layer (EGL) of 23-week human fetal cerebellum relative to the other MB variants (Figure 2B). Interestingly, CD171 exhibited a nodular staining pattern in some areas of SHH tumor samples, while displaying uniformly positive staining throughout the other 3 variants as well as in 23-week human fetal cerebellum (Figure 2C). In contrast, CD106 expression was restricted to small foci of neurons in the fetal cerebellum and was not detectable in the MB samples (Figure 2D). It should be noted that 3 of the 25 cell surface makers from our Lyoplate ™ screens (GD2, SSEA4, and CD57), are not proteins and therefore, would not be represented in the transcriptome datasets. Therefore, we evaluated expression levels of these markers by flow cytometry in cell lines. Although GD2 levels were much higher in self-renewing *vs*. non-self-renewing cells (Table 1), GD2 levels varied in tumorspheres from all the cell lines tested. CD57 levels were inconsistent, and SSEA4 showed negligible expression in all cell lines examined (data not shown).

Collectively, our results suggest that CD271 and CD171 are the best candidates for additional functional testing. Importantly, these data validate our previous findings demonstrating higher expression of CD271 in stem/progenitor SHH Daoy MB cells [18]. As CD271 was strongly upregulated in SHH cell culture and patient samples relative to the other variants, we have chosen to pursue CD271 as our lead candidate for functional characterization.

### CD271 overexpression results in a decrease in tumorsphere number but an increase in tumorsphere size over subsequent passage *in vitro*

**Figure 3:**
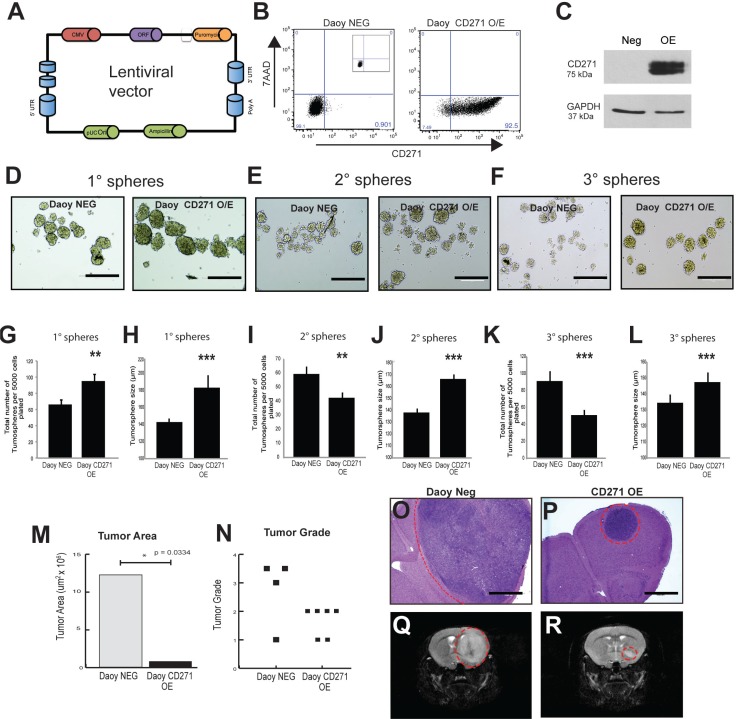
CD271 overexpression changes the size and number of Daoy tumorspheres **A.** pReceiver-Lv105 lentiviral construct used for stable overexpression of CD271 in Daoy cells. **B.**-**C.** Validation of CD271 OE in Daoy cells by flow cytometry (B) and Western blot (C). GAPDH serves as a loading control. **D.**-**F.** Representative images of primary (D), secondary (E), and tertiary (F) tumorspheres from Daoy negative control cells and stable Daoy CD271 OE cells. **G.**-**H.** Primary tumorsphere number (G) and tumorsphere size (H) are increased in Daoy OEs *vs*. controls **I.**-**L.** Tumorsphere number is decreased in secondary (I) and tertiary (K) Daoy OE cells compared to controls; whereas tumorsphere size is increased in secondary (J) and tertiary Daoy CD271 OEs (L). Error bars: s.e.m. *P* < 0.01**, *P* < 0.001 ***. **M**.-**N**. Injection of CD271 OE cells into NOD SCID mice results in tumors with a (M) significantly smaller tumor area, *p* = 0.0334, and a lower grade (N) *p* = 0.1461, than cells expressing lower, endogenous levels of CD271. **O.**-**P.** Representative H&E staining of tumors from mice injected with Daoy control cells (O) and CD271 OE cells (P). **Q.**-**R.** Representative MRI imaging of mice injected with Daoy control cells (Q) and CD271 OE cells (R).

To gain further insight into the functional role of CD271 in SHH MB cells, we generated a stably overexpressing CD271+ line from adherent SHH Daoy MB cells using lentiviral constructs (Figure 3A-3B). The Daoy line is traditionally cultured in serum, and under these differentiated conditions, CD271 levels are negligible [18]. However, when Daoy cells are adapted to tumorsphere culture in stem cell medium as shown in our flow cytometry screens and analyses, CD271 levels increase [18]. As this is a dynamic process, we wanted to test the effects of constitutive CD271 overexpression in SHH MB cells. Following stable selection, CD271 overexpression (OE) was validated by flow cytometry and Western Blot (Figure 3B-3C). To measure self-renewal capacity, tumorsphere assays were performed over 3 passages. CD271 OE resulted in a significant increase in tumorsphere number, and tumorsphere size compared with the negative controls (Figure 3D, 3G-3H). Following passage to secondary spheres (P2), we observed a decrease in sphere number (Figure 3E, 3I); however, the larger tumorsphere size was maintained in CD271 OE cells relative to controls (Figure 3J). The same patterns were seen following passage to tertiary (P3) tumorspheres (Figure 3F, 3K-3L). There was an increase in total cell counts in P1 tumorspheres (Figure S3A), however there was no significant difference in P2 and P3 tumorspheres (Figure S3B-S3C) or viability as measured by Trypan blue staining (Figure S3D-S3F). In support of our total cell count data, expression of cell cycle genes (*CDK2*, *CDK6*, *CCND1*, *CCND2*) as measured by qPCR showed no significant difference between CD271 OE and control tumorspheres (Figure S3G). Staining for the proliferation marker Ki67 showed similar results as there was no difference in the frequency of positive cells (Figure S3H)

We next evaluated tumor cell invasion in CD271 OE cells relative to controls using a hanging drop assay followed by implantation of aggregates into collagen gels [38]. Following 3 days invasion, there was no significant difference in CD271 OE vs. control cells (Figure S3I-S3J). Collectively, the loss of tumorsphere number over passage and maintenance of larger tumorsphere size suggest that stable overexpression of CD271 may regulate the self-renewing phenotype in SHH MB.

### CD271 overexpression results in smaller tumors *in vivo*

Based on our *in vitro* overexpression data, we hypothesize that CD271 is selecting for a lower self-renewing stem or progenitor cell in SHH MB. Both cell types are potential cells of origin for this molecular variant[13, 14, 39, 40]. A decreased self-renewal capacity in CD271 OE cells, irrespective of whether selection is for a stem or progenitor cell, may result in decreased tumor growth following injection of CD271 OE cells *in vivo*. To test this hypothesis, 5 × 10^4^ CD271 OE Daoy MB passage 1 tumorspheres (*N* = 6) and their controls (*N* = 4) were xenografted into the cerebral cortex of NOD SCID mice and evaluated after 13 weeks. Immunohistochemical staining revealed sustained overexpression of CD271 *in vivo* (Figure S3K-S3N). Indeed, cells stably overexpressing CD271 formed tumors; however, they were significantly smaller as demonstrated by a decreased tumor area (Figure 3M) and lower tumor grade (Figure 3N), when compared to control cells expressing lower endogenous levels of CD271. Control cells formed very large tumors in the striatum and thalamus (Figure 3O, 3Q), whereas CD271 OE cells formed masses consisting of small tumor deposits in the striatum (Figure 3P, 3R). These *in vivo* results support our *in vitro* findings and suggest that constitutive CD271 overexpression may lead to selection of a progenitor or lower self-renewing stem cell.

### Compound X γ-secretase inhibitor decreases tumorsphere size and increases tumorsphere number *in vitro*

**Figure 4:**
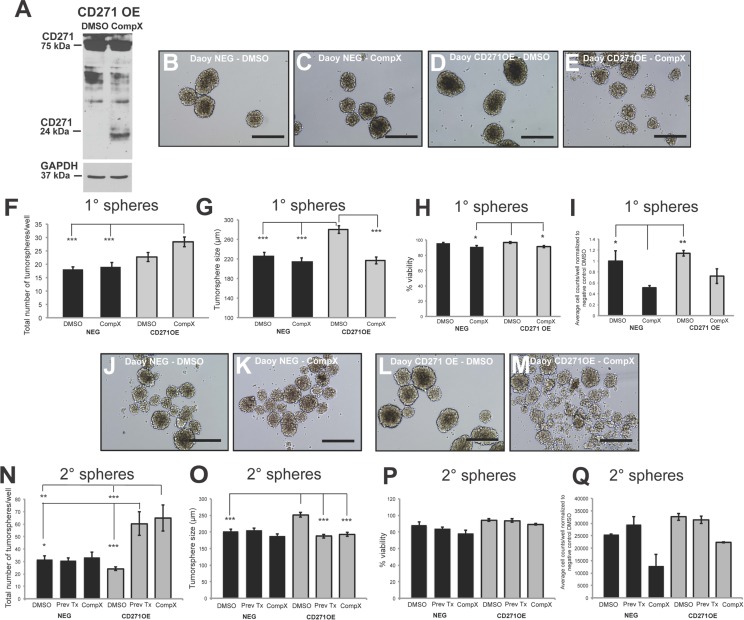
γ- secretase inhibitor (Compound X) treatment of Daoy CD271 OE cells results in a reversal of the OE phenotype **A.** Western blot demonstrating C-terminal fragment accumulation upon blocking of CD271 cleavage by the γ- secretase inhibitor, Compound X. **B.**-**E.** Representative images of primary Daoy NEG (B-C) and Daoy CD271 OE (D-E) tumorspheres following treatment with Compound X. Scale bar: 400 μm. **F.**-**G.** Treatment of Daoy NEG and Daoy CD271 OE primary tumorspheres with Compound X results in an increase in sphere number (F) and decrease in sphere size (G). **H.**-**I.** Quantification of cell viability (H) and total cell number (I) in Daoy NEG and Daoy CD271 OE primary tumorspheres following treatment with Compound X. **J.**-**M.** Representative images of secondary Daoy NEG (J-K) and Daoy CD271 OE (L-M) tumorspheres following treatment with Compound X. Scale bar: 400 μm. **N.**-**O.** Treatment of Daoy NEG and Daoy CD271 OE secondary tumorspheres with Compound X either in passage 1 and 2 or in passage 1 only (Prev Tx). **P.**-**Q.** Quantification of cell viability (P) and total cell number (Q) in Daoy NEG and Daoy CD271 OE secondary tumorspheres following treatment with Compound X. Error bars: s.e.m. *P* < 0.05*, *P* < 0.01**, *P* < 0.001 ***.

We next sought to determine whether proteolytic processing of CD271 is required for the effects on tumorsphere size and self-renewal in SHH MB cells. CD271 has been shown to undergo intramembrane proteolysis, where the extracellular domain is cleaved by a α-secretase, followed by γ-secretase cleavage of the C-terminal fragment to release the intracellular domain [41]. This process appears to be associated with a variety of functions including invasion in adult glioma brain tumors and proliferation in glioma brain tumor initiating cells [42, 43]. Blocking the proteolytic process by γ-secretase inhibitor has been shown to reduce MB spinal metastasis [44]. Although the γ-secretase inhibitor has additional targets such as the Notch pathway and is not specific to CD271, it is worthwhile to evaluate the effect of this compound, as, to our knowledge, it has not been tested on stem/progenitor SHH MB cell phenotypes. To determine the effect of a γ-secretase inhibitor on SHH MB cells expressing high levels of CD271, we treated the CD271 stably overexpressing Daoy cells with Compound X. CD271 OE cells treated with 2 μM Compound X γ-secretase inhibitor exhibit an accumulation of the 24 kDa C-terminal fragment band, consistent with previous studies [44] indicating a blockage of γ-secretase-mediated processing of CD271 relative to the DMSO control (Figure 4A). After treatment with Compound X, CD271 OE cells generated significantly smaller tumorspheres than DMSO treated CD271 OE cells. There was no significant difference in tumorsphere size between Compound X and DMSO treated negative control cells (Figure 4B-4G). This reduction in size was maintained through secondary tumorspheres and was also accompanied by a significant increase in tumorsphere number when compared to DMSO treated cells (Figure 4J-4O). Overall, no differences were observed for viability or cell counts following treatment with Compound X (Figure 4H-4I, P-Q). Interestingly, secondary tumorspheres derived from cells treated with Compound X in passage 1 and vehicle only in passage 2 (“previously treated”) also showed significantly reduced tumorsphere size and increased tumorsphere number, similar to those tumorspheres treated with Compound X over both passages.

**Figure 5:**
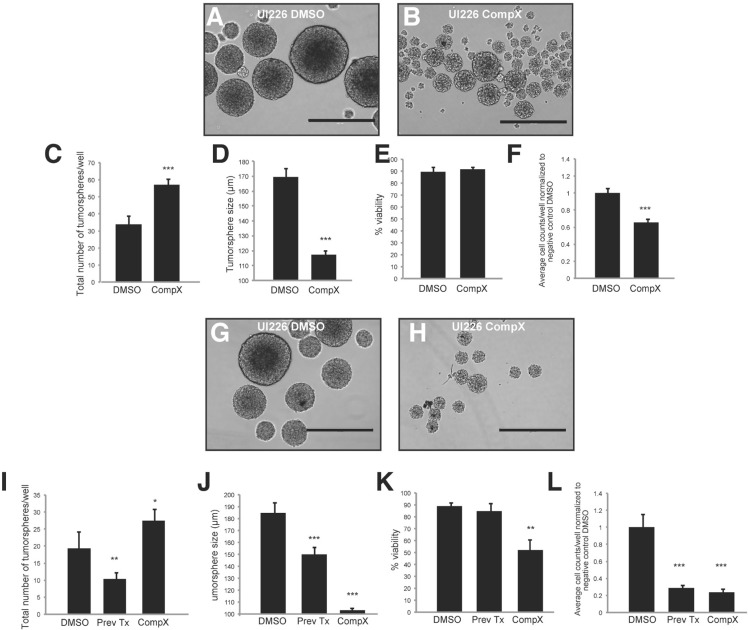
Treatment of UI226 tumorspheres with Compound X over 2 passages **A.**-**B.** Representative images of primary UI226 tumorspheres following treatment with DMSO (B) or Compound X (C). Scale bar: 400 μm. **C.**-**D.** Treatment of UI226 primary tumorspheres with Compound X results in an increase in total sphere number (C) and decrease in sphere size (D). **E.**-**F.** Quantification of cell viability (E) and total cell number (F) in UI226 primary tumorspheres following treatment with Compound X. **G.**-**H.** Representative images of secondary UI226 tumorspheres following treatment with DMSO (G) or Compound X (H). Scale bar: 400 μm. **I.**-**J.** Treatment of UI226 secondary tumorspheres with Compound X either in passage 1 and 2 or in passage 1 only (Prev Tx) results in an increase in sphere number (I) and decrease in sphere size (J). **K.**-**L.** Quantification of cell viability (K) and total cell number (L) in UI226 secondary tumorspheres following treatment with Compound X. Error bars: s.e.m. *P* < 0.01**, *P* < 0.001 ***.

Similar results were obtained with UI226 primary SHH MB cells that are nearly 100% for CD271 (Figure 2A) After treatment with Compound X, we observed a significant increase in the total number of tumorspheres (Figure 5A-5C) concomitant with a decrease in UI226 tumorsphere size relative to DMSO treated cells (Figure 5A-5B, 5D). We did not observe changes in cell viability; however, there was a significant decline in cell number in passage 1 spheres (Figure 5E-5F). Secondary tumorsphere numbers were also significantly increased concomitant with a decreased tumorsphere size (Figure 5G-5J). Similar to Daoy CD271 OE cells, secondary tumorspheres derived from cells treated with Compound X in passage 1 (“previously treated”) also showed a decrease in tumorsphere size (Figure 5J); however, in this case, the increased number of spheres was not sustained and was actually lower compared with DMSO controls (Figure 5I). In addition, cell viability was not significantly different, while the total number of cells decreased (Figure 5K-5L). For UI226 cells, Compound X treatment may need to be sustained over subsequent passage to continually affect all cell properties in tumorsphere culture. Collectively, these results demonstrate that blocking of CD271 processing by γ-secretase inhibitor reverses the OE phenotype observed in SHH MB cells.

### CD271 knockdown results in generation of smaller tumorspheres *in vitro*

**Figure 6:**
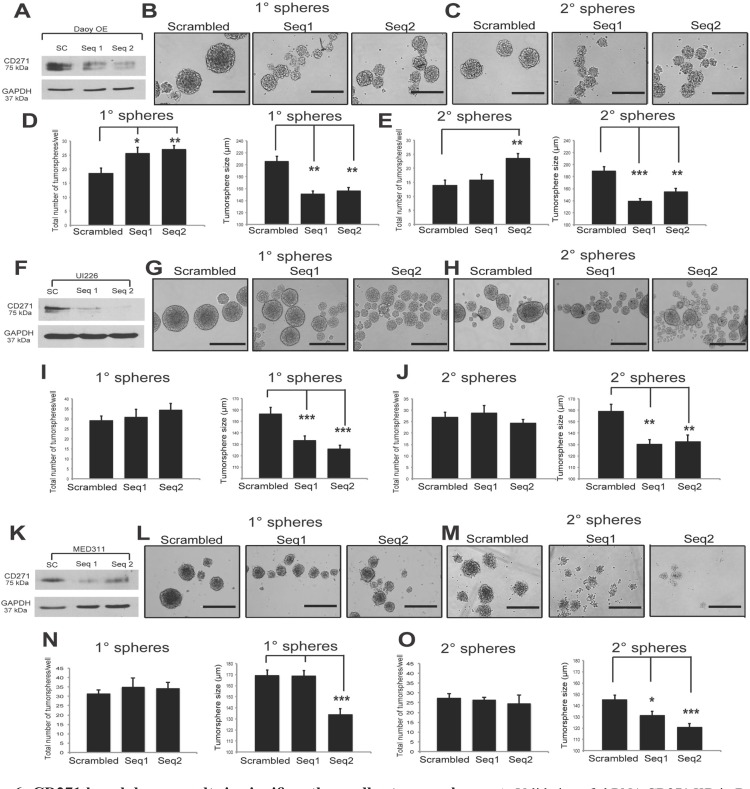
CD271 knockdown results in significantly smaller tumorspheres **A.** Validation of shRNA CD271 KD in Daoy OE cells by Western blot. GAPDH serves as a loading control. **B.**-**C.** Representative images of primary (B) and secondary (C) tumorspheres from Daoy OE cells infected with scrambled negative control *vs*. stable CD271 KD from 2 independent shRNA sequences. **D.**-**E.** Quantification of primary (D) and secondary (E) tumorsphere number and size following CD271 KD in Daoy CD271 OE cells. **F.** Validation of shRNA CD271 KD in UI226 cells by Western blot. GAPDH serves as a loading control. **G.**-**H.** Representative images of primary (G) and secondary (H) tumorspheres from UI226 cells infected with scrambled negative control *vs*. stable CD271 KD from 2 independent shRNA sequences. **I.**-**J.** Quantification of primary (I) and secondary (J) tumorsphere number and size following CD271 KD in UI226 cells. **K.** Validation of shRNA CD271 KD in MED311 cells by Western blot. GAPDH serves as a loading control. **L.**-**M.** Representative images of primary (L) and secondary (M) tumorspheres from MED311 cells infected with scrambled negative control *vs*. stable CD271 KD from 2 independent shRNA sequences. **N.**-**O.** Quantification of primary (N) and secondary (O) tumorsphere number and size following CD271 KD in MED311 cells. Error bars: s.e.m. *P* < 0.05*, *P* < 0.01**, *P* < 0.001 ***.

To complement our CD271 overexpression studies and to look at specificity of CD271 using a more direct method, we generated CD271 knockdown (KD) cells from our CD271 OE line as well as MED311-FH and UI226 SHH cells which, as opposed to Daoy, were originally derived and cultured in stem-cell propagating conditions and therefore express very high endogenous levels of CD271 (Figure 2A). Two different shRNA sequences targeting CD271 were used, along with a scrambled negative control. Both sequences resulted in a KD of CD271 compared to scrambled in all cells tested (Figure 6A, 6F, 6K). Similar to the effects seen in our γ-secretase inhibitor treatment, CD271 KD in Daoy OE cells exhibited an increase in tumorsphere number and decrease in tumorsphere size compared to controls in P1 and P2 tumorspheres (Figure 6B-6E). CD271 KD in MED311 and UI226 cells also results in a decrease in P1 and P2 tumorsphere size compared to controls (Figure 6G-6J, 6L-6O); however, no significant changes in tumorsphere number were observed (Figure 6I-6J, 6N-6O). We did not observe consistent changes in total cell counts and viability as measured by Trypan blue staining in Daoy OE (Figure S4A-S4D), UI226 (Figure S4E-S4H) or MED311 cells (Figure S4I-L). Cell cycle gene (*CDK2*, *CDK6*, *CCND1*, *CCND2*) expression was also measured by qPCR in UI226 and MED311KD cells (Figure S4M-S4N), and the differences were either not significant or inconsistent between the 2 shRNA sequences; however, there was a trend towards increased expression of *CCND1* and *CCND2* in UI226 cells. These KD data provide additional support for our overexpression studies and suggests that CD271 plays a role in regulating the SHH MB stem/progenitor cell state.

### CD271 levels are inversely correlated with expression of SHH signaling pathway genes

**Figure 7:**
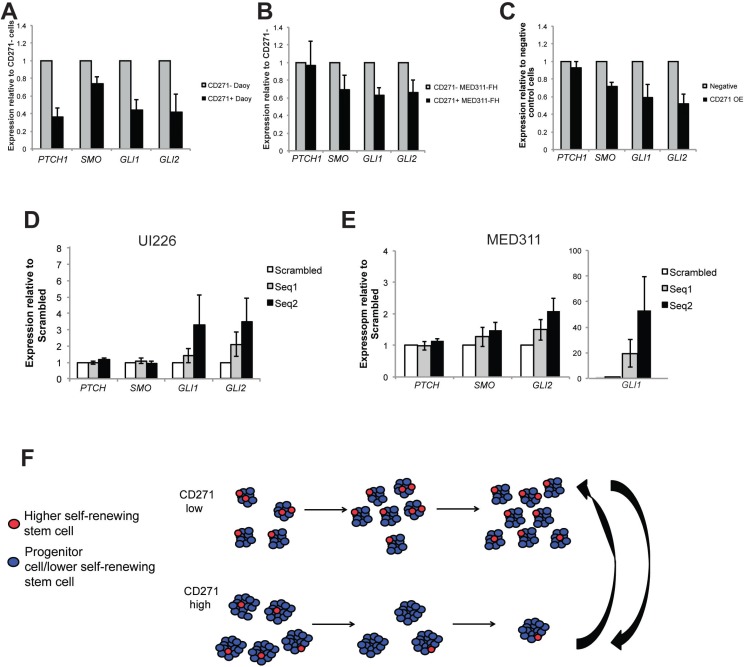
CD271 levels are inversely correlated with expression of SHH pathway genes **A.**-**C.** qPCR analysis of *PTCH*, *SMO*, *GLI1* and *GLI2* gene expression in Daoy sorted CD271+ *vs*. CD271− cells (A), MED311 sorted CD271+ *vs*. CD271− cells (B), and Daoy OE *vs*. negative control cells (C). (**D.**-**E.)** qPCR analysis of *PTCH*, *SMO*, *GLI1* and *GLI2* gene expression in UI226 (D) and MED311 (E) cells following knockdown of CD271. Error bars: s.e.m. *P* < 0.01**, *P* < 0.001 ***. **F.** Model depicting the role of CD271 in selecting for a lower self-renewing progenitor or stem cell phenotype in SHH MB cells.

Since we are utilizing a SHH MB variant for our studies and SHH pathway genes have been shown to be upregulated in CD133+ putative stem cell populations in Daoy cells [24], we examined expression of the SHH signaling pathway genes in CD271+ vs. CD271− cells and in CD271 OE *vs*. control cells by qPCR. We observed a significant downregulation of Patched (*PTCH*), Smoothened (*SMO*), *GLI1*, and *GLI2* in Daoy sorted CD271+ compared to CD271− cells and a significant downregulation of *GLI1* and *GLI2* in MED311 sorted CD271+ vs. CD271− cells (Figure 7A-7B). qPCR analysis of SHH pathway genes in CD271 OE cells compared to controls also revealed a downregulation of *SMO*, *GLI1*, and *GLI2* (Figure 7C). In contrast, following knockdown of CD271 in MED311 and UI226 cells, we typically observed an increase in expression of the downstream SHH pathway genes, *GLI1* and *GLI2,* for both cell lines (Figure 7D-7E). These results demonstrate that higher CD271 levels are negatively correlated with expression of SHH signaling pathway genes.

## DISCUSSION

In this study, we utilized an integrated approach of high throughput flow cytometry screening combined with gene expression profiling to identify cell surface markers that are differentially expressed in self-renewing *vs*. non-self-renewing SHH MB cells as well as in SHH MB *vs*. other MB variants. The Lyoplate platform has been utilized for characterizing specific cell phenotypes in multiple cancers [19, 21]; however, this technique has not been used in pediatric cancers such as MB. While this study focused on characterization of CD271, our results also show that CD171 is a candidate for additional functional testing, as this cell surface marker has been linked with tumor progression, metastasis and therapeutic resistance in a wide variety of cancers [45-53].

We have demonstrated a novel role for CD271 in regulating the SHH MB stem/progenitor cell state and present a working model whereby CD271 is associated with a dynamic SHH MB progenitor or lower self-renewing stem cells within a heterogeneous tumor population (Figure 7F). CD271 is a transmembrane glycoprotein that plays a variety of roles in normal neurodevelopment including growth cone elongation, axon guidance, cell survival and cell death, depending on the cellular context [22]. The four CD271 ligands, NGF, BDNF, NT-3 and NT-4 exist in both pro-neurotrophin form and mature form, and this results in different effects depending on the presence/absence of ligand as well as which ligand is bound [22]. For example, CD271 has been shown to induce apoptosis in the absence of ligand or binding of an unprocessed neurotrophin (pro-neurotrophin) [54]. Activation of the JNK pathway appears to play a role in CD271 mediated cell death, whereas signaling through the NFκB pathway promotes survival [54]. Future studies will determine which pathways are regulated by CD271 in SHH MB progenitors and stem cells. In addition to its role in neurodevelopment, CD271 has been shown to regulate adult glioblastoma brain tumor invasion and has been shown to be a selective tumor propagating cell marker in melanoma, esophageal squamous cell carcinoma, and hypopharyngeal cancer [42, 55]. Importantly, CD271 has also been shown to mark a neurogenic precursor population, which includes stem and progenitor cells, in the subventricular zone (SVZ) from both rats and mice [56]. Using FACS to isolate CD271+ postnatal rat SVZ, the authors showed that sorted cells with the highest levels of CD271 generated the most neurospheres. They also demonstrated that CD271 regulates neurogenesis and the ongoing generation of olfactory bulb neurons in the SVZ [56].

Similarly, our results suggest that CD271 selects for a stem/progenitor cell population in tumorsphere assays. However, there is conflicting evidence in the literature regarding the interpretation of sphere size changes and multiple explanations have been proposed [57]. For example, larger spheres have been positively correlated with the number of stem cells and capacity for self-renewal. However it is also possible that size is merely related to differentiation potential, proliferation of progenitor cells and/or response to growth factors [57]. Thus, larger spheres may actually contain more progenitors as opposed to stem cells. In our model systems, we did not observe any consistent changes in proliferation; however, future studies will evaluate the effect of CD271 on differentiation of SHH MB cells in the presence of its ligands. Given the difficulties in interpreting sphere size data, we have not distinguished between a stem or progenitor cell in our gain/loss of function studies. CD271 was typically associated with an overall lower self-renewal capacity but an increased sphere size.

Studies conducted in mouse models have shown that the SHH variant of MB arises from granule precursor cells in the external granular layer (EGL) of the cerebellum [39, 40]. In addition, Li et al. showed that a population of nestin-expressing progenitor cells, distinct from granular neuroprogenitor cells in the EGL, are responsible for tumorigenesis [58]. CD271 immunostaining in paraffin embedded sections of 23-week human fetal cerebellum (Figure 2B) revealed high expression of CD271 in the EGL, an area with a high density of progenitor cells, and is consistent with staining profiles previously observed [59, 60], In MB, previous work has demonstrated conflicting roles for CD271 in MB apoptosis [61, 62]; however, in both cases, experiments were conducted with D283, or non-SHH MB cells, further underscoring the need to evaluate the function of a cell surface marker in both a subtype-specific and cell context-dependent manner.

Activation of the SHH signaling pathway is characteristic of SHH MB. Studies utilizing SHH pathway inhibitors have shown that patients display an initial response, however this is followed by eventual relapse attributed to drug resistance [63-66]. Zhukova et al. recently shed light on this issue by demonstrating that *P53* mutations are associated with poor outcome for SHH patients, and this may account for treatment failure within this subgroup [4]. Intratumoral heterogeneity likely contributes to SHH pathway inhibitor resistance. Wang *et al.* [24] also provided insight into SHH MB heterogeneity by showing elevated expression of SHH pathway genes and increased sensitivity to pathway inhibition in the CD133+ stem cell fraction in Daoy cells. In contrast, we demonstrated CD271+ cells, a cell population previously shown to be mutually exclusive to CD133+ cells [18], exhibit a down regulation of SHH pathway genes. As such, CD271+ cells may be less responsive to SHH pathway inhibition emphasizing the importance of understanding tumor heterogeneity when attempting to dissect the complex factors leading to targeted therapy. In support of this concept, Chow et al. [67] recently demonstrated that *Patched* (*Ptch*) +/− Shh MB tumors are composed of 3 cell types, each with unique cellular and molecular properties in tumorsphere culture. These cell types also exhibited different response to cyclopamine, a SMO inhibitor, with growth factor dependent, as opposed to growth factor independent, cultures displaying resistance to this SHH pathway inhibitor.

The characterization and subsequent functional validation of novel cell surface markers in pediatric brain tumors has clinical implications. Gene expression profiling and genomic sequencing are powerful techniques for delineating the dysregulated pathways driving tumorigenesis; however, these methods do not preserve cellular integrity or function. Characterization of the cell surface proteome enables researchers to employ strategies for isolation of specific cellular phenotypes that can then be utilized for additional molecular, functional and most importantly pre-clinical testing *in vitro* and *in vivo.* Moreover, subtype specific biomarkers can be useful as a diagnostic tool. For instance, the specificity of CD271 expression in SHH tumors makes it an interesting candidate for simple and fast diagnostic screening for this subtype using flow cytometry or immunohistochemistry immediately after tumor resection. Indeed, our results are consistent with previous findings demonstrating high CD271 expression levels in tumors specifically with desmoplastic histology that typically belong to the SHH subgroup [60, 61]. We have used cell surface marker screening as a component of a systematic discovery platform to show that MB stem/progenitor marker profiles are not universal but can be subtype specific across all 4 molecular variants. Future studies will evaluate the value of CD271 as a potential diagnostic marker for SHH MB cells using a large cohort of patient samples.

Current treatment for MB involves surgery, chemotherapy and radiation which mainly target the proliferating cell population and tumor mass [68]. Identification of tumor propagating cell populations for specific MB molecular variants will enable isolation of a novel cell resource for design of next generation targeted therapies that target both tumor stem cell and progenitor populations. This can only be achieved through combinatorial therapy targeting multiple cell populations. While SHH pathway antagonists may be ideal for targeting a portion of the stem cell population, additional treatment would be required to selectively target the CD271+ progenitor or stem cell fractions that are in a lower self-renewing state. The Compound X γ-secretase inhibitor treatment and CD271 knockdown studies support the notion that targeting one cell population merely shifts cells towards a different phenotype and would therefore not be effective in completely eradicating the tumor. Nevertheless, CD271 serves as a potential therapeutic target as there is no detectable expression of CD271 in the brains of children or adults [59]. Thus, treatment aimed at CD271 and its downstream targets would ultimately lessen the broad impact of toxic treatments such as radiation and chemotherapy on the child's developing nervous system and improve the quality of life for those who survive long-term.

## MATERIALS AND METHODS

### Culture of cell lines and primary MB cells

Daoy human MB cells (originally derived from a desmoplastic cerebellar MB[23]), D341, and D283 were purchased from the American Type Culture Collection (ATCC, Rockville, MD, USA). D341 [34] was utilized as a representative of Group 3 MB. D283 cells [35] have been classified as Group 4 [36]; however, other studies suggest that this cell line is Group 3 given the high c-myc levels [37]. Daoy cells were cultured in Eagle's minimum essential media (EMEM) (ATCC) containing 10% FBS (Fisher Scientific, Ottawa, Ontario). Upon reaching confluency, cells were dissociated in Accutase (Invitrogen, Burlington, ON, Canada) and passed 1:10. Daoy MB subclones were derived by single cell deposits into 96-well plates using flow cytometry as described [18]. MED311-FH cells were obtained from Dr. James Olson (Fred Hutchinson Cancer Research Center) and have been subtyped as SHH. MED311-FH cells were cultured in NeuroCult proliferation medium (Stem Cell Technologies, Vancouver, BC, Canada) on laminin-coated plates (BD Biosciences, San Jose, CA, USA). Upon reaching confluency, cells were dissociated in Accutase and passed 1:4. D283 were cultured as adherent cultures in EMEM containing 10% FBS. D341 were cultured in ultra low attachment plates, with DMEM/F12 containing 15 B27, 1% N2, 20 ng/ml EGF, and 20 ng/ml bFGF (neural precursor medium).

UI226 cultures were established under an IRB approved protocol by the Central Nervous System Tissue Bank, Department of Neurosurgery, University of Iowa, and obtained through a material transfer arrangement by Translational Genomics, Inc. UI226 cells are SHH MB as analyzed by NanoString [33] and were originally passaged ( < 10 times) in nude mice as flank injections. UI226 cells were then adapted to cell culture in StemPro^®^ Neural Stem Cell Serum Free Medium (Life Technologies, Burlington, ON, Canada) on laminin-coated plates (BD Biosciences). UI226 cells cultured as tumorspheres in ultra low attachment plates were also grown in StemPro^®^ medium.

### Tumorsphere assay

Cells were dissociated and aliquots of 2500 or 5000 cells from Daoy CD271 OE cells, Daoy CD271 KD and their respective controls were plated in 24-well ultra-low attachment plates in Neural Precursor Media. 2500 cells/well were used for tumorsphere size counts whereas 5000 cells/well were used for tumorsphere number and total cell counts. MED311 CD271 KD cells were also dissociated and 1×10^4^ cells per well were plated in the same conditions as mentioned above. For UI226 KD cells, dissociated cultures were re-plated in ultra-low attachment plates at 5000 cells/well. Cells were incubated for 5 days, after which tumorspheres were counted and measured. Tumorspheres from each population were then dissociated and re-plated in aliquots of the respective cell number for secondary and subsequent tertiary tumorsphere assays. Secondary and/or tertiary tumorspheres were counted at day 5.

### High throughput flow cytometry screening

Flow cytometry was performed using BD Lyoplate Human Screening Panels (BD Biosciences) consisting of 242 cell surface markers and 9 isotype controls. Tumorspheres from Daoy subclones were dissociated and resuspended in Dulbecco's phosphate-buffered saline (DPBS) (Fisher Scientific, Ottawa, ON, Canada) containing 0.5% FBS. 2 × 10^4^ cells were plated in 96 well plates in a total volume of 100 μl. Ten microliters of diluted antibody was added to each well and incubated for 20 minutes on ice. Cells were washed twice using 0.5% FBS/PBS. Fifty microliters of secondary goat anti mouse or goat anti rat antibody was added to cells and incubated on ice for 20 minutes. Cells were then washed twice more using FBS/PBS and 0.5 ul of 7AAD was then added to the cell suspensions as an indicator of cell viability. Cells were then analyzed using the Guava easyCyte flow cytometer (EMD Millipore, Etobicoke, ON, Canada). Results were analyzed and compiled using FlowJo software and exported to a Microsoft Excel 2007 template for generation of heat maps. This enables side-by-side comparative analysis of multiple screens from different cell types. CD271, CD106, CD171 and EGFR levels were validated in Daoy subclones, MED311, UI226, D283, and D341 cells cultured as tumorspheres in ultralow attachment plates with neural precursor medium. On day 4, tumorspheres were fed by removal and replacement of 1 ml of medium. On day 7, Daoy tumorspheres were dissociated, washed, and resuspended in DPBS containing 0.5% FBS. Cells were then stained with one of the following antibodies: CD271, CD106, CD171, and EGFR. All antibodies were obtained from BD Biosciences. Flow cytometry was performed on the Gallios Flow Cytometer (Beckman Coulter, Mississauga, ON, Canada) and analyzed using Kaluza software (Beckman Coulter).

### Cell surface marker profiling in MB transcriptome datasets

CD271 (p75/NTR), CD171 (L1CAM), EGFR, and CD106 (VCAM1) expression levels were examined in 3 independent previously described transcriptome datasets comprising 548 patient samples (Boston (*N* = 199 samples using Affymetrix_HT- HG-U133A chips [32]), Heidelberg (*N* = 64 samples using Agilent Whole Human Genome Oligo Microarrays [31]), and Toronto (*N* = 285 samples using Affymetrix Gene 1.1 ST Arrays [30])). Expression of the 4 markers across all samples was presented in boxplot format as log2-transformed signal intensity. All subgroups were compared using a Kruskal-Wallis test for significance.

### Medulloblastoma patient sample subgrouping

Samples were obtained in accordance with the local research ethics board at the University of Manitoba. Additional samples were obtained from the Brain Tumour Tissue Bank (Brain Tumour Foundation of Canada, London Health Sciences Centre, London, Ontario, Canada). Total RNA was extracted from 3-5 paraffin scrolls using the Qiagen RNeasy FFPE kit and 200ng of total RNA was analyzed by NanoString as previously described [33]. Subgroup determination was performed in the R statistical environment (v3.1.2) as previously described by PAM prediction using the pamr package (v1.55). A total of 10 samples were subtyped and utilized for immunohistochemistry: 1 WNT, 6 SHH, 1 Group 3 and 2 Group 4.

### Immunohistochemistry

Paraffin embedded tissue from patient samples as well as 23-week human fetal cerebellum was deparaffinized in xylene and processed through a graded series of alcohol concentrations. Antigen retrieval was performed at 95-100°C for 20 minutes in Citrate Buffer pH 6.0. Samples were blocked with 3% lamb serum in 1XPBS (for CD271) or 5% Goat serum / 1% BSA (for CD171 and CD106) in TBS and subsequently treated with primary antibody diluted in 1% lamb serum in 1XPBS (for CD271) or 1% goat serum / 1% BSA in TBS (for CD171 and CD106) overnight at 4°C: CD271: (1:400) (Millipore, Etobicoke, Ontario, Canada), CD171 (1:150) (Biolegend, San Diego, CA, USA), CD106/VCAM-1 (1:500) (abcam, Cambridge, MA, USA). Slides were washed in (CD271): 1XPBS, (CD171/CD106): 1XTBS and treated with secondary antibody: for CD271 (1:500), for CD106 (1:200) (Biotin-SP-Affinipure Goat Anti-Rabbit IgG (Jackson Immunoresearch, West Grove, PA, USA) and for CD171 (1:200) (Biotin-SP-AffiniPure Sheep Anti-Mouse IgG (H+L) (Jackson Immunoresearch) diluted in (CD271): 1% lamb serum in 1XPBS, (CD171/CD106): 1% goat serum / 1% BSA diluted in TBS for 2 hours at room temperature. Slides were treated with 1:400 dilution of Streptavidin/HRP (Jackson Immunoresearch) in (CD271): 1XPBS (CD171/CD106): 1XTBS for 30 min and subsequently developed using DAB. Slides were counterstained with hematoxylin. Coverslips were mounted with Permount (Fisher Scientific).

### Lentiviral infection

Stable overexpression (OE) of CD271 was generated by infecting Daoy cells with the pReceiver-Lv105 lentiviral construct (GeneCopoeia, Rockville, MD, USA) containing a puromycin resistance gene. Lentifect™ Lentiviral Particles were used as a negative control. Puromycin was used for selection and was replenished every 3 days. CD271 OE was assessed by Western blot and flow cytometry. CD271 was stably knocked down in Daoy OE, UI226, and MED311 cells using 2 shRNAmir constructs (Open Biosystems, Inc., Thermo Fisher, Huntsville, AL, USA) consisting of a dual expression system with TurboGFP as a transduction marker. A non-silencing (scrambled) shRNA sequence was used as a negative control. CD271 knockdown (KD) was assessed by Western blot. Following stable selection, Daoy OE KD, UI226 KD, MED311 KD, and their respective controls were subjected to tumorsphere assays.

### Invasion assay

Cells were dissociated and aliquots of 2.5 × 10^4^ cells were prepared as hanging drops in 20 μl as described [38]. Hanging drops were incubated for 3 days to form aggregates and then transferred to collagen type I gels (VWR, Mississauga, ON, Canada) prepared as previously described. Following collagen gelation at 37°C, embedded aggregates were then overlain with EMEM containing 10% FBS. Aggregate measurements were taken at day 0 and invasion was measured at 72 hours (day 3) using a Zeiss Primo Vert microscope (Carl Zeiss Canada Ltd., Toronto ON, Canada) with micrometer.

### γ-secretase inhibitor treatment

Daoy CD271 OE and control cells were plated in neural precursor medium in 24 well ultra-low attachment plates and media was supplemented with 2 μM Compound X γ-secretase inhibitor (EMD Millipore) or DMSO (vehicle control). UI226 cells were cultured as tumorspheres and grown in StemPro^®^ media supplemented with 2 μM Compound X γ-secretase inhibitor or DMSO. For both cell lines, tumorspheres were counted, measured and passed to secondary tumorspheres in media supplemented with either 2 μM Compound X γ-secretase inhibitor or DMSO. After 5 days, Passage 2 tumorspheres were counted and measured.

### Quantitative reverse transcription-polymerase chain reaction

Daoy, UI226 and/or MED311 tumorspheres were dissociated, and counted using an automatic cell counter and stained for CD271. Sort samples were also stained with 7AAD viability dye (Beckman Coulter). Cells were sorted on the basis of CD271+ and CD271− expression using a MoFlo XDP cell sorter. Analysis of cell sorting was performed using FlowJo software. Total RNA was extracted from Daoy control, CD271 OE, sorted Daoy CD271+/−, sorted MED311 CD271+/− cells, as well as MED311 and UI226 KD cells using the Norgen All in one kit (Norgen Biotek, Thorold, ON, Canada) according to manufacturer's guidelines. First-strand cDNA was synthesized using the Superscript III First Strand Synthesis System (Life Technologies)). The following PCR conditions were used: 50°C for 2 minutes, 95°C for 2 minutes, and 40 cycles of 95°C for 15 seconds and 60°C for 30 seconds. qPCR was conducted using GoTaq qPCR Master Mix (Fisher Scientific) and performed on an Mx3000P (Stratagene, Santa Clara, CA, USA) qPCR system. All values were normalized to glyceraldehyde 3-phosphate dehydrogenase (GAPDH). Specific primer sequences for each gene are listed in Table S1.

### Western blot

Protein was isolated from all cells and their respective controls using the All-In-One Purification Kit (Norgen Biotek) according to manufacturer's instructions. Twenty micrograms of protein from Daoy CD271 OE, CD271 OE/KD cells and their controls were separated by SDS PAGE using 10% acrylamide gels. Forty micrograms of protein from MED311 KD, UI226 KD and controls, 10 μg of γ-secretase treated CD271 OE, and 40 μg of γ-secretase treated UI226 along with control cells were also separated by SDS PAGE. Protein was transferred using a semi-dry transfer method to nitrocellulose membrane (BioRad). Membranes were blocked in 5% non-fat milk in Tris Buffered Saline with Tween 20 (TBST) and then incubated at 4°C overnight with antibodies to CD271 (Millipore, 1:1000) and CD271-ICD (Promega, 1:500). Membranes were washed several times with TBST before application of goat anti-rabbit horseradish peroxidase secondary antibody (BioRad Laboratories Ltd, Mississauga, ON, Canada,1:3000). Membranes were developed using SuperSignal West Pico (Fisher Scientific).

### Intracerebral transplantations and histology

The University of Manitoba Animal Care Committee approved all procedures and protocols. Daoy control and Daoy CD271 OE tumorspheres were intracerebrally injected into non-obese diabetic severe combined immunodeficient (NOD/SCID) mice as previously described [11, 18, 69]. Briefly, mice were anesthetized with isoflurane and injected in the right frontal lobe with biologic replicates consisting of 5 × 10^4^ for Daoy control and CD271 OE tumorspheres. After 12 weeks, animals were perfused with formalin and the brains were extracted, placed in formalin for 2 to 7 days, embedded in paraffin, and then sectioned (5-μm thickness). Sections were de-waxed in xylene and rehydrated through a graded series of alcohol concentrations. Samples were stained with hematoxylin and eosin. Slides were mounted and imaged using an EVOS xl core microscope (AMG, Seattle, WA, USA). Malignant cell growth was scored, from a scale of 0 to 4 in arbitrary units, where 0 = no malignant cells with certainty, 1 = indicates rare clusters of malignant cells confined to subarachnoid compartment, 2 = malignant cells in subarachnoid compartment and infiltrating perivascular spaces, and 3 = features in 2, in addition to tumor nodules growing in other areas of the brain or cerebellum. For each tumor sample, two slides containing six to seven brain sections were scored and averaged to obtain a grade. Slides were assessed by a neuropathologist who was blinded to cellular identity.

Stained slides were also scanned using an Aperio ScanScope CS slide scanner (Leica Microsystems Inc., Concord, ON, Canada) at 20x maximum magnification. Tumor area was measured using Aperio ImageScope software (Leica Microsystems Inc., Concord, ON.). Tumor was freehand selected (Figure S5) and calculated using area measure function. Total area was measured in 6 slices, each 2 mm in distance apart, representing the anterior to posterior region of brain. Total tumor area for each sample was calculated by adding tumor area in all the slices.

### Magnetic resonance imaging

Mice were anesthetized using 5% isoflurane in O_2_/N_2_O and maintained at 2% isoflurane in O_2_/N_2_O with a nose cone. Respiration and external body temperature were monitored during imaging using an MR-compatible small animal monitoring and gating system (SA Instruments, Stony Brook, NY, USA). External body temperature was maintained at 37°C with a heating circulator bath (Thermo Scientific Haake, Karlsruhe, Germany). Mouse heads were held in place with a tooth bar inside a custom-built 24 mm diameter, 300 MHz inductively coupled quadrature RF volume coil (NRC Institute for Biodiagnostics, Winnipeg, MB, Canada). The entire apparatus was placed inside a Bruker BGA12-S actively shielded gradient system with integrated shim coils (Bruker BioSpin, Milton, ON, Canada). All MR experiments were performed on a 7 T 21 cm Bruker Avance III NMR system with Paravision 5.0 (Bruker BioSpin). The mouse brain was imaged in prone position rostral to caudal using 12 slices with a slice thickness of 0.75mm and an interslice distance of 1.0mm.

### Statistical analysis

All tests were performed using Prism 5 software (GraphPad Software, La Jolla, CA, USA) or SPSS Statistics (IBM, Armonk, NY, USA). Descriptive statistics were used to determine significant differences including mean and SEM along with one-way analyses of variance (ANOVA), independent sample two-tailed *t* tests, and Tukey's test for multiple comparisons. P values less than .05 were considered significant.

## SUPPLEMENTARY MATERIAL FIGURES AND TABLE


