# Supplementary material for: Characterization of novel biomarkers in selecting for subtype specific medulloblastoma phenotypes
Source: Oncotarget. 2015 Oct 20;6(36):38881–900. doi: 10.18632/oncotarget.6195 (PMC4770744; doi:10.18632/oncotarget.6195)
Supplement: Supplementary file 1 [file oncotarget-06-38881-s001.pdf]

Characterization of novel biomarkers in selecting for subtype specific medulloblastoma phenotypes

Supplementary Material

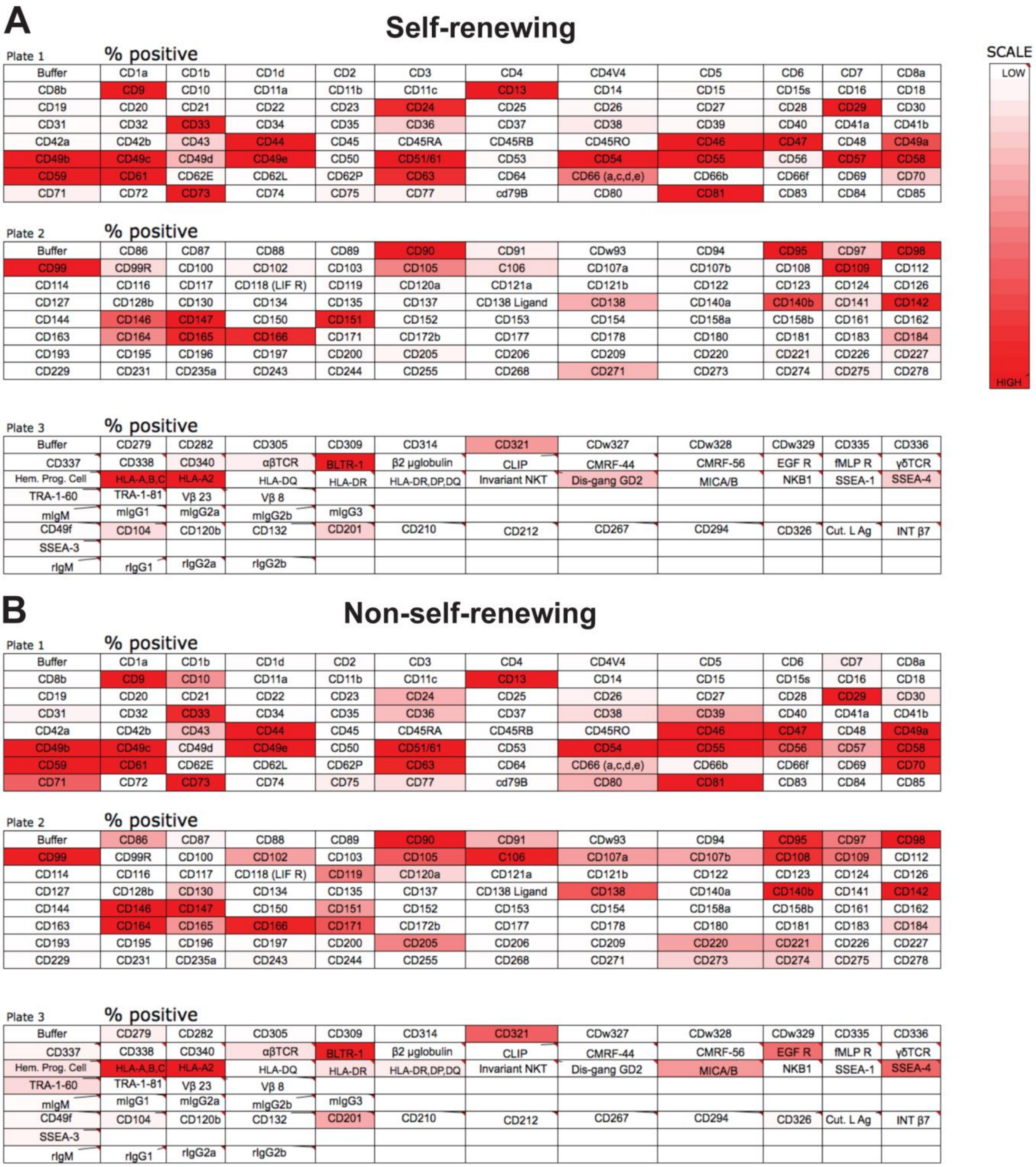

Supplemental Figure 1: Heat maps showing frequencies of cell surface markers in self-renewing (SR) tumorspheres (A) and non-self-renewing (NSR) tumorspheres (B) from the Daoy cell line. Red indicates high expression and white indicates negligible or absent expression.

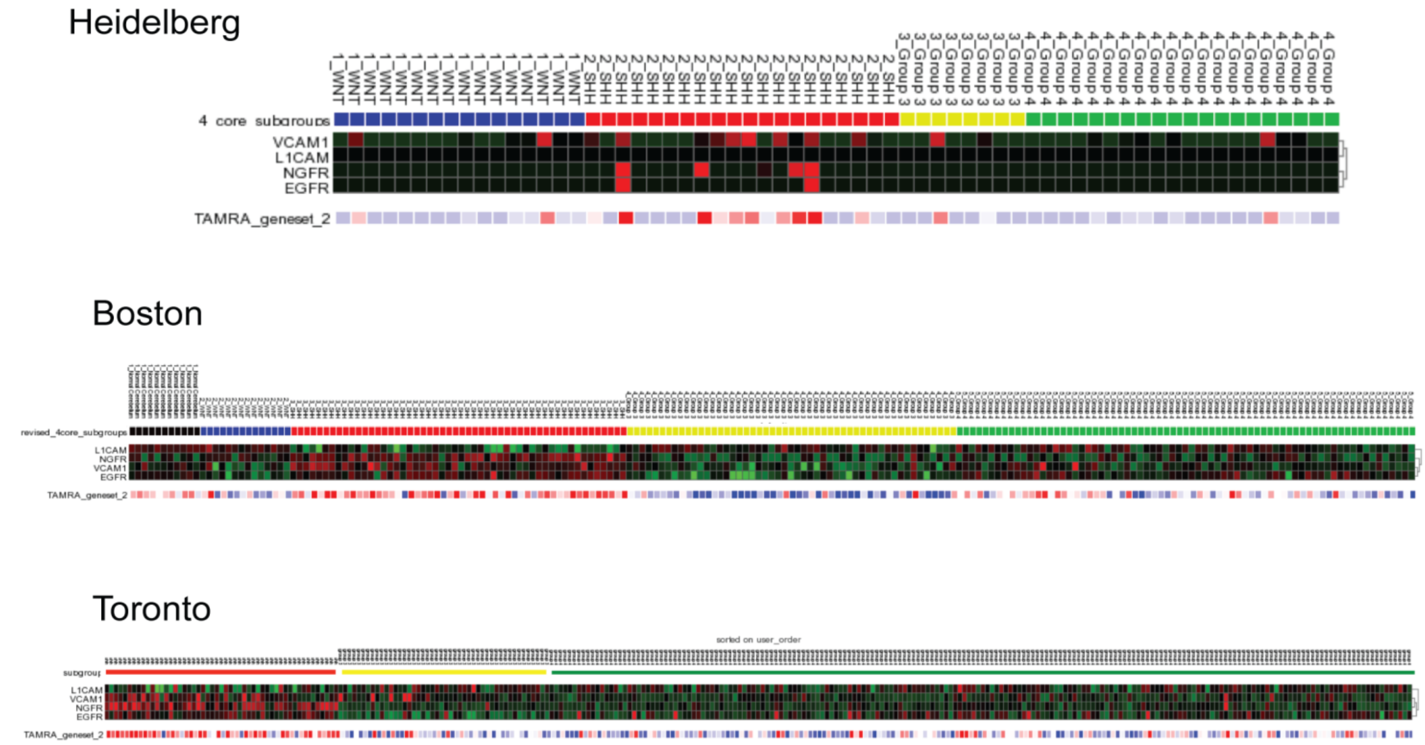

Supplemental Figure 2: Validation of candidate biomarkers using gene expression profiling. Gene expression profiling data from 3 independent datasets representing 548 patient samples showing relative enrichment of CD106/VCAM1, CD271/p75NTR and EGFR and downregulation of CD171/NCAM-L1 in SHH tumors compared with the other variants.

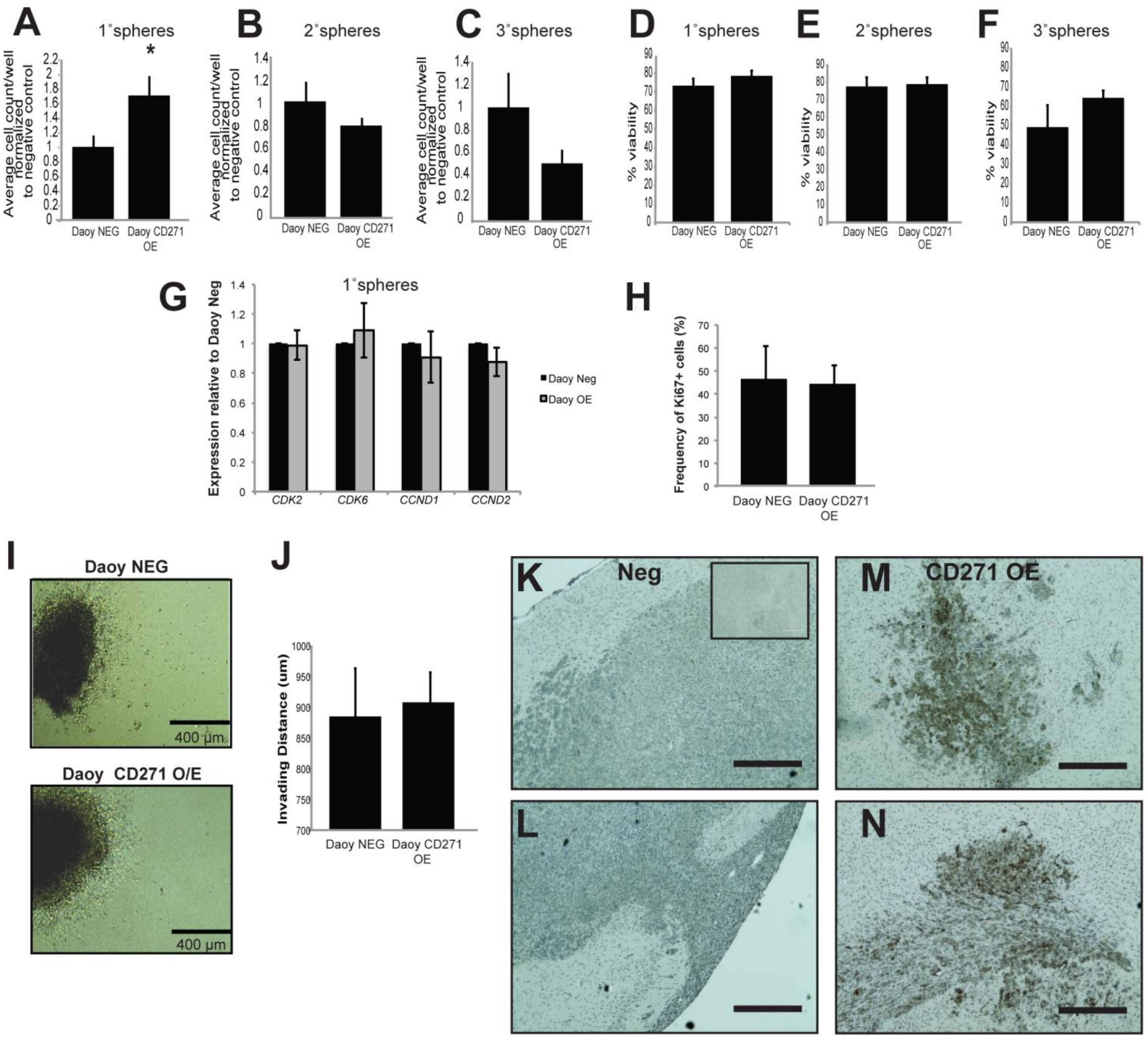

Supplemental Figure 3: Total cell counts, viability and invasion following CD271 OE in Daoy cells A-C. Average cell count/well normalized to negative control for Daoy CD271 OE tumorspheres. **D-F.** Quantification of cell viability in primary (D), secondary (E), and tertiary (F) CD271 OE tumorspheres vs. negative control. **G.** qPCR analysis of *CDK2, CKD6, CCND1,* and *CCND2* gene expression in Daoy OE vs. negative control P2 tumorspheres. **H.** Frequency of Ki67+ cells in Daoy NEG and Daoy CD271 OE P2 tumorspheres. **I-J.** Representative images (I) and quantification (J) of invasion for Daoy NEG and Daoy CD271 OE aggregates in a collagen matrix. Error bars: s.e.m. P<0.05*. **K-N.** Immunohistochemistry staining for CD271 in xenografts derived from Daoy NEG (K,L) and Daoy CD271 OE (M,N) tumorsphere cells injected intracranially into NOD SCID mice. Inset: secondary only negative control. Scale bar: 400 μm.

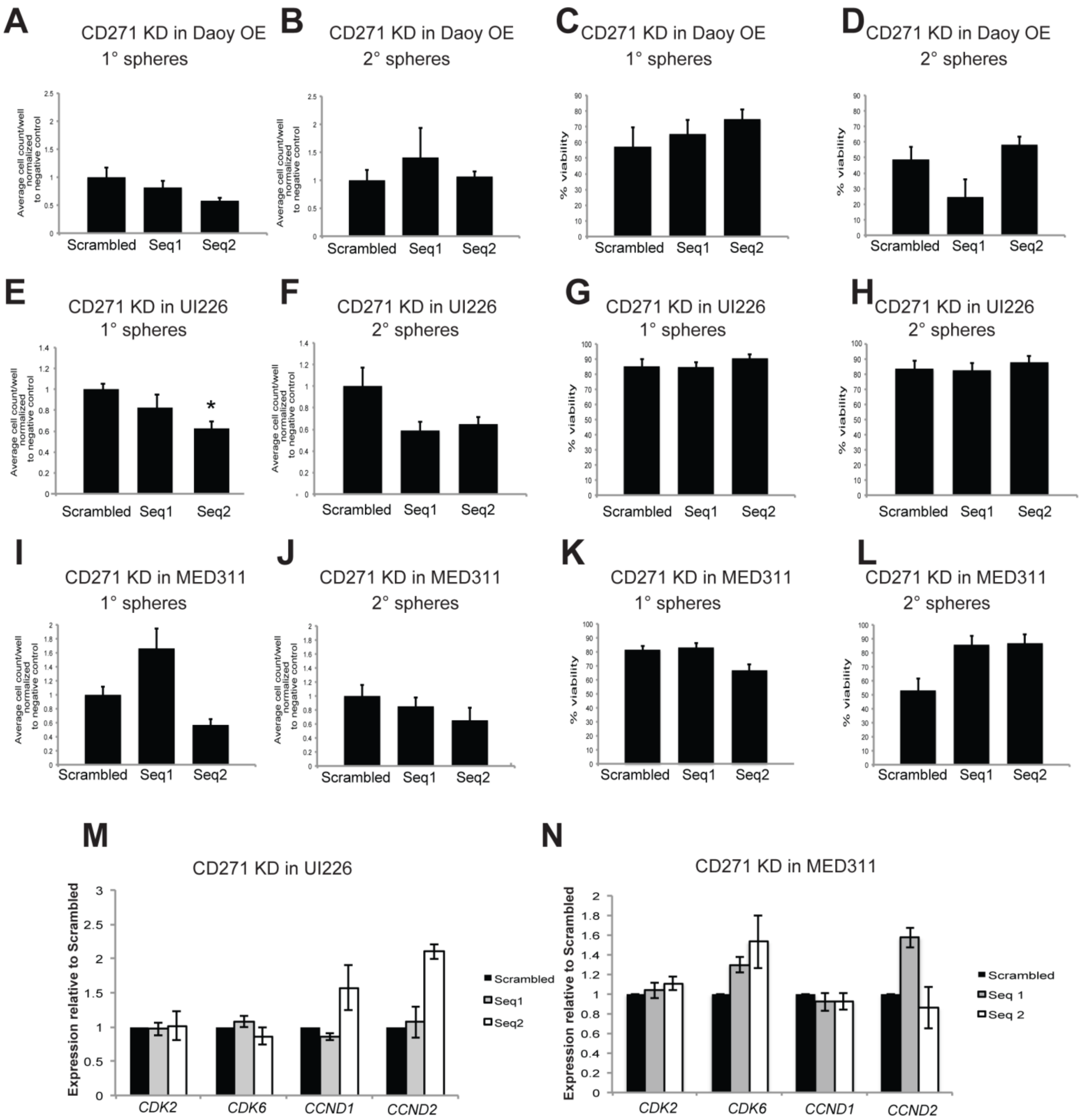

Supplemental Figure 4: Total cell counts and viability are not significantly different following CD271 knockdown. A-B. Average cell count/well normalized to negative control (scrambled) for CD271 KD in Daoy OE primary (A) and secondary (B) tumorspheres. **C-D.** Percent viability for CD271 KD in Daoy OE primary (C) and secondary (D) tumorspheres. **E-F.** Average cell count/well normalized to negative control (scrambled) for CD271 KD in UI226 primary (E) and secondary (F) tumorspheres. **G-H.** Percent viability for CD271 KD in UI226 primary (G) and secondary (H) tumorspheres. **I-J**. Average cell count/well normalized to negative control (scrambled) for CD271 KD in MED311 primary (I) and secondary (J) tumorspheres. **KL.** Percent viability for CD271 KD in MED311 primary (K) and secondary (L) tumorspheres. **M-N.** qPCR analysis of *CDK2, CKD6, CCND1, CCND2* gene expression in (M) UI226 and (N) MED311 KD vs. scrambled control P1 tumorspheres. Error bars: s.e.m.

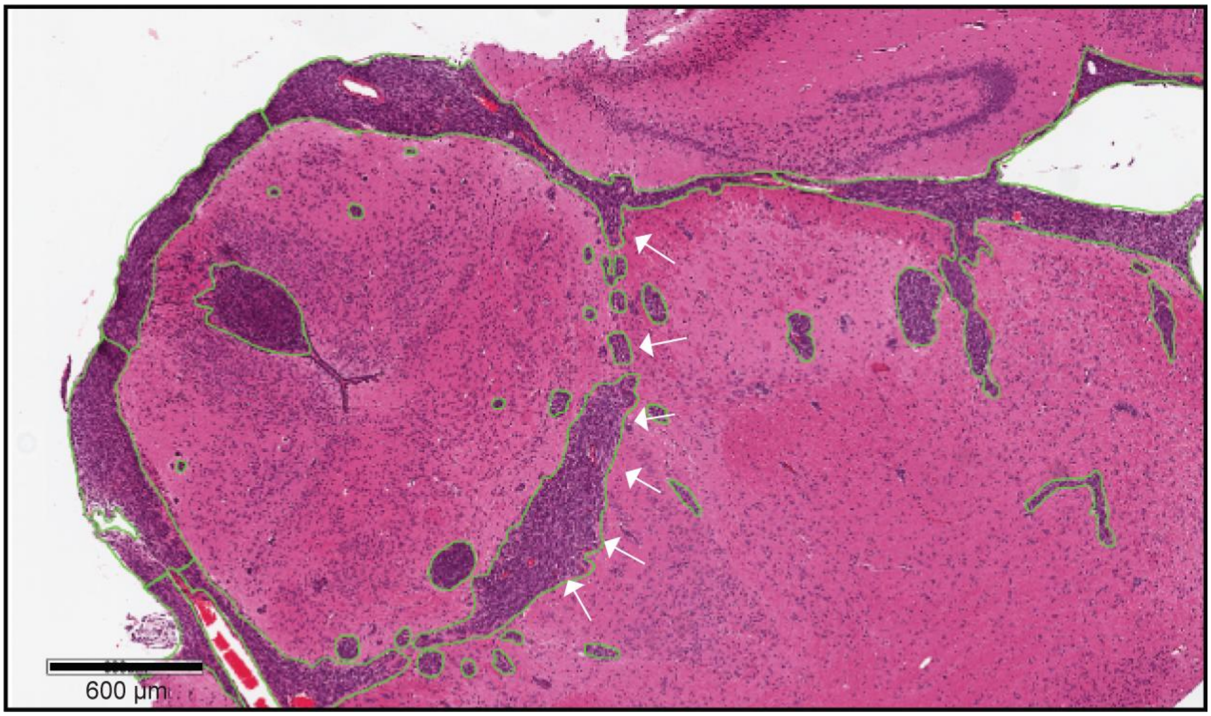

Supplemental Figure 5: Representative image of labeled tumor sections for quantification of area. Representative image of tumor area measurement calculated from 6 slices, each 2 mm in distance apart, spanning the anterior to posterior region of brain. Tumor was freehand selected and calculated using area measure function. Total tumor area for each sample was calculated by adding tumor area in all slices. Arrows denote tracing path of tumor area.

Supplemental Table 1. Primer sequences used for qPCR reactions

Gene	Forward sequence	Reverse Sequence
CD271 (*NGFR*)	5'-AGG CTG GGC CGA CGC CGA GTG-3'	5'-GGG GCT GGG-AGC TGC CCA TCA-3'
PTCH	5'-GGG AGG AAA TGC TGA ATA AGG-3'	5'-TCT GGA TAA GCC ATG ACA TCC-3'
SMO	5'-TTT GGC TTT GTG CTC ATT ACC-3'	5'-CAT GGC AAA CAG GTT GAT CTT-3
GLI1	5'-CCC AAG GCT CTA GGT GGA AC-3'	5'-CTG GGG GTA ATG GGA AAA GA-3'
GLI2	5'-CAG CAT CTC TTG CCA CCA TT-3'	5'-AAG CCG GAT CAA GGA GAT GT-3'
CDK2	5'-TGC CTG ATT ACA AGC CAA GTT-3'	5'-TAG GGT CGT AGT GCA GCA TTT-3'
CDK6	5'-TTC CAG ATG GCT CTA ACC TCA-3'	5'-AAT ATG CAG CCA ACA CTC CAG-3'
CCND1	5'-TCT ACA CCG ACA ACT CCA TCC-3'	5'-CTG GCA TTT TGG AGA GGA AGT-3'
CCND2	5'-GCT GGC TAA GAT CAC CAA CAC-3'	5'-AGT TCA TCC TCC GAC TTG GAT-3'
